# Low-Loss Paper-Substrate Triple-Band-Frequency Reconfigurable Microstrip Antenna for Sub-7GHz Applications

**DOI:** 10.3390/s23218996

**Published:** 2023-11-06

**Authors:** Ajit Kumar Singh, Santosh Kumar Mahto, Rashmi Sinha, Mohammad Alibakhshikenari, Ahmed Jamal Abdullah Al-Gburi, Ashfaq Ahmad, Lida Kouhalvandi, Bal S. Virdee, Mariana Dalarsson

**Affiliations:** 1 Indian Institute of Information Technology, Ranchi 834010, India; 2 National Institute of Technology, Jamshedpur 831014, India; 3 Department of Signal Theory and Communications, Universidad Carlos III de Madrid, 28911 Leganés, Madrid, Spain; 4 Center for Telecommunication Research & Innovation (CeTRI), Fakulti Teknologi dan Kejuruteraan Elektronik dan Komputer (FTKEK), Universiti Teknikal Malaysia Melaka (UTeM), Ayer Keroh 75450, Malaysia; 5 Chemistry Department, College of Science, King Saud University, P.O. Box 2455, Riyadh 11451, Saudi Arabia; 6 Department of Electrical and Electronics Engineering, Dogus University, Istanbul 34775, Turkey; 7 Center for Communications Technology, London Metropolitan University, London N7 8DB, UK; 8 Department of Electrical Engineering, School of Electrical Engineering and Computer Science, KTH Royal Institiute of Technology, 100 44 Stockholm, Sweden

**Keywords:** Internet of Things (IoT), multiband antenna, patch antenna, PIN diodes, reconfigurable antenna

## Abstract

In this paper, a low-cost resin-coated commercial-photo-paper substrate is used to design a printed reconfigurable multiband antenna. The two PIN diodes are used mainly to redistribute the surface current that provides reconfigurable properties to the proposed antenna. The antenna size of 40 mm × 40 mm × 0.44 mm with a partial ground, covers wireless and mobile bands ranging from 1.91 GHz to 6.75 GHz. The parametric analysis is performed to achieve optimized design parameters of the antenna. The U-shaped and C-shaped emitters are meant to function at 2.4 GHz and 5.9 GHz, respectively, while the primary emitter is designed to operate at 3.5 GHz. The proposed antenna achieved peak gain and radiation efficiency of 3.4 dBi and 90%, respectively. Simulated and measured results of the reflection coefficient, radiation pattern, gain, and efficiency show that the antenna design is in favorable agreement. Since the proposed antenna achieved wideband (1.91–6.75 GHz) using PIN diode configuration, using this technique the need for numerous electronic components to provide multiband frequency is avoided.

## 1. Introduction

The rapid growth of technology requires significant innovations to upgrade the present devices to meet the demands of present and new wireless communications and Internet of Things (IoT) applications. An antenna plays a vital role in wireless communication, so with advancement in technology the antenna needs to be modified. Antenna designs with fixed characteristics can be replaced by evolvable antennas that provide multi-band and reconfigurable characteristics that can be used for many applications [1]. Small antennas can contribute to meeting the modern requirements of wireless communications, as well as IoT applications. Microstrip antennas have been popular so far, since they are easy to produce and design, while offering a limited bandwidth. With the remarkable progress of wireless communication technologies, the use of reconfigurable antennas instead of traditional antennas could be the best solution for multiple applications providing multi-band frequencies. Reconfigurable antennas can dynamically change their operating frequency, pattern, or polarization to suit different communication requirements. This adaptability is particularly valuable in scenarios where the operating environment or frequency bands can change rapidly. While active components like RF switches and tunable elements can achieve reconfigurability in traditional antennas, they add complexity, power consumption, and cost. Reconfigurable antennas, when designed effectively, can achieve similar functionality with fewer components, leading to simpler and more reliable systems. Since the last decade, reconfigurable antennas have been designed using thick [2], RT Duroid [3], PCB [4], silicon [5], FR-4 [6,7], RO4003 (thick) [8], and organic materials [9,10] such as Liquid Crystal Polymer (LCP), Iraqi palm-tree-remnant (IPTR) substrates and Polyethylene terephthalate (PET).

If the above mentioned substrates are replaced with a paper substrate, then the cost is reduced by a factor of ten [11]. Besides being low-cost, paper substrates are light weight and provide feasibility testing. Some antennas have recently been designed on paper substrate [12,13,14,15,16], but none of them are reconfigurable. To achieve the antenna reconfigurability, the use of physical, mechanical, and optical switches are required [8]. Different types of electronic switches have been used earlier by research groups such as Micro-electromechanical systems (MEMS) [5,9], but these switches have a few drawbacks as they require high DC-bias voltages (40–100 V), for which they need costly transformers that make the system expensive. The use of a complementary metal oxide semiconductor (CMOS) [13] is available at a low cost and consumes less power, but with a high propagation delay. Compared to all other switches, the use of PIN diodes [17] provides a low insertion loss, low cost, and good isolation that makes it beneficial for the reconfigurability of the antenna by changing the switching states alternatively. Various inkjet-printed antennas have been studied recently for multiple technologies [18]. In reference [19], the utilization of a 3D parasitic layer allows for antenna geometry manipulation via switches. However, the complexity of such a three-dimensional structure poses challenges in terms of fabrication and integration into an IoT device. For 4G and early 5G applications, a frequency reconfigurable MIMO antenna was introduced in [20]. Two radiating/receiving elements were arranged in an array, and placed diagonally across from one another in the design. Two PIN diodes, functioning in dual-frequency modes (2.4 GHz and 3.5 GHz), were employed, with two V-shaped protrusions on the patch forming a hybrid configuration of a frequency- and pattern-reconfigurable antenna that has been presented [21]. The defective ground structure (DGS) phenomenon, which is prominent in the bandwidth of 3200 MHz from 2.7 GHz to 5.9 GHz and provides a gain of 3.1 dBi, was presented to expand the bandwidth of the suggested antenna. A low-profile antenna was suggested in [22] to change the radiation pattern and frequency. The substrate was the Rogers RT Duroid 5870, which has a dielectric constant of 2.33. Frequency reconfiguration can be achieved by linking patches of different lengths that align with their respective resonant frequencies, using three PIN diode switches. A transparent flexible co-planar waveguide fed patch antenna, using polyethylene terephthalate substrate, was presented in [23]. A semi-flexible 2 × 1 array antenna was proposed using epoxy-glass fiber and graphene as the patch and ground, respectively, in [24]. A microstrip patch antenna with a center parasitic patch of a half wavelength, and a slot in the radiating patch have been incorporated for the bandwidth enhancement in the order of 79.56% (2.21–5.13 GHz).

In the present study, a low-cost adhesive copper-on-paper substrate is constructed as a reconfigurable antenna that can be used for IoT applications, wireless communication, and mobile bands. The antenna’s main radiator is made up of a variety of smaller radiators that each support a distinct frequency band. These shapes are designed to minimize the overall size of the antenna and to have a multiple-band response. The copper material as the radiating element and the PIN diode as the switching circuits are printed on the paper substrate, which makes it convenient and smooth for the integration of antenna into the IoT device. The antenna is designed on a paper substrate with a thickness of 0.44 mm, a dielectric constant of 3.2, and a loss tangent of 0.05 for multiple applications, and providing triple-band frequency ranges 1.91–2.68 GHz, 3.33–3.75 GHz, and 5.55–6.75 GHz. The frequency band changes with the change in switching state of the PIN diodes. By altering their switching conditions, the PIN diodes found in the antenna construction contribute significantly to the achievement of various frequency bands. The advantage of the proposed design is using photo paper as a substrate. The realization of the multi-band antenna on a low-cost paper substrate using adhesive copper has the advantages of a small size, low profile, and simple configuration. The low profile of the antenna makes it a promising candidate for future compact and slim wireless devices.

## 2. Design

**Figure 1:**
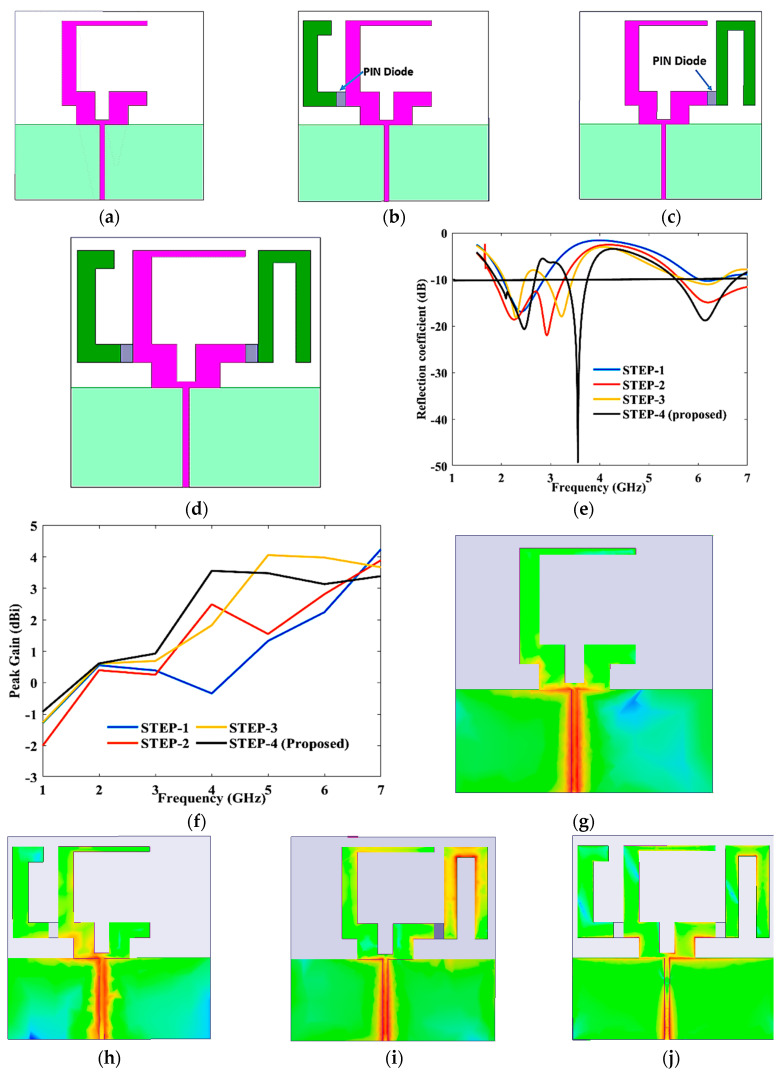
The evolution of the proposed antenna: (**a**) Step-I; (**b**) step-II; (**c**) step-III; (**d**) step-IV; and (**e**) reflection coefficient. (**f**) Peak gain and current distributions; (**g**) step-I-2.4 GHz; (**h**) step-II-2 GHz; (**i**) step-III-3.2 GHz; and (**j**) step-IV-5.6 GHz.

Figure 1 shows the evolution of the proposed design of antenna. In step-I, a modified C-shaped radiator is used with a partial ground plane, as shown in Figure 1a. The required S11 is achieved at a lower frequency (1.9–2.7 GHz), as shown in Figure 1e. A C-shaped radiator is then connected to the main radiator with PIN diode (D1), as shown in Figure 1b; a dual band is achieved (1.9–3.1 GHz), and (5.5–6.8 GHz) but it excludes the band 3.3–3.7 GHz. Further, an inverted U-shaped radiator is connected in step-I, as shown in Figure 1c; the radiator achieved triple-band response but very low return loss along with a narrow band, as shown in Figure 1e. The primary radiator is finally connected to both (U-shaped and C-shaped) in step-IV using two PIN diodes, as shown in Figure 1d. The proposed antenna achieved triple-band response in the ranges of 1.91–2.68 GHz, 3.33–3.75 GHz, and 5.55–6.75 GHz, as can be seen in Figure 1e. Figure 1f shows the peak gain of antenna evolution steps. From Figure 1f, it is visible that through using auxiliary C- and U-shaped radiators peak gain has increased in the operating-frequency range. In step-II, peak gain is lower, as compared to step-I in lower frequency range, because of current distribution that is contradicting the main radiator. Similarly, Figure 1g–j represent the surface-current distribution of antenna-evolution steps at multiple frequency. A 2 GHz current concentrated more at the feed of the patch, and a 5.9 GHz current concentrated more at the edge of the patch and auxiliary radiator.

**Figure 2:**
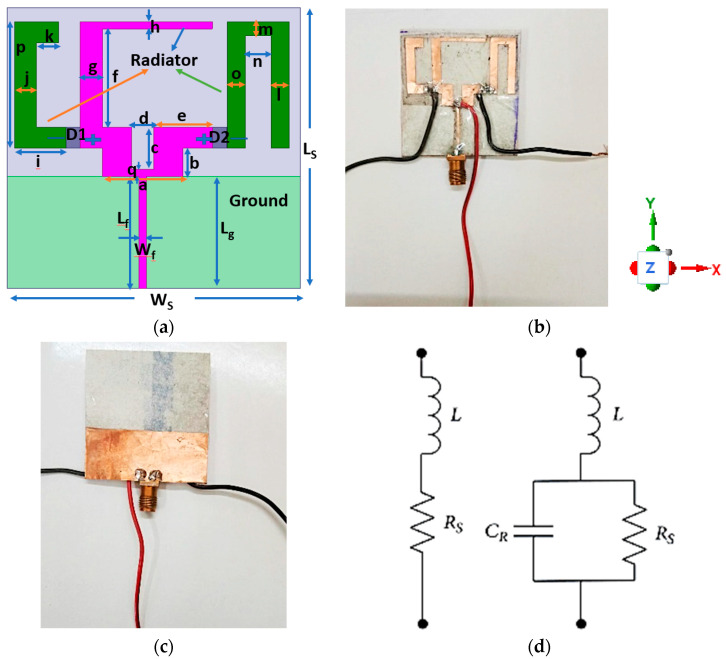
(**a**) Proposed antenna (layout- simulated) and fabricated antenna; (**b**) front view; (**c**) back view; and (**d**) equivalent circuits for the PIN diode ON and OFF condition.

The suggested microstrip antenna is depicted in Figure 2a, in both front and side views. It is built on a substrate made of inexpensive resin-coated photo paper [1]. Figure 2b,c shows the front view and back view of the fabricated antenna design. Photo paper was one of the best choices for flexible antennas due to the low cost of the substrate, and wearable properties; this paper antenna can be used for a reasonable amount of time and can then be disposed off, making it highly suitable for wireless applications. The ground plane is placed on the bottom flat sheet, while the antenna is placed on the top flat sheet. Instead of conductive ink, adhesive copper was utilized to emphasize the paper’s dielectric properties. The conductivity and thickness are not constant, and this leads to fluctuating conductive losses that are difficult to accurately account for in simulations. The antenna and the ground plane of the antenna are printed on two sheets of photo paper, each with a thickness of 0.22 mm. Using two sheets help to reach a reasonable thickness (0.44 mm). Alignment marks have been implemented on the photo papers so that the two photo papers can be glued together with correct alignment to form a single antenna substrate.

The proposed antenna, which lacks a ground plane directly beneath the substrate, presents different design considerations compared to, for instance, a patch antenna with a ground plane directly beneath it. In this context, the chosen antenna design is significantly influenced by the choice of substrate. Altering the substrate material would have a substantial impact on the antenna’s performance.

Changing the substrate can affect the antenna in several ways, including the following: 

**Size and Dimensions:** The physical dimensions of the antenna, including the size and shape of the radiating element, are influenced by the substrate. Changing the substrate might require adjustments to the antenna’s physical design.

**Resonant frequency:** The substrate material affects the antenna’s resonant frequency. A change in the substrate can shift the operating frequency of the antenna, which may or may not align with the intended frequency band.

**Operating bandwidth:** The choice of substrate can affect the antenna’s bandwidth. A new substrate may widen or narrow the antenna’s operational bandwidth.

**Radiation pattern:** The substrate influences the radiation pattern of the antenna. Different substrates can lead to variations in the antenna’s radiation characteristics, such as beamwidth and directivity.

**Efficiency:** The substrate’s dielectric properties impact the antenna’s efficiency. A different substrate can lead to changes in radiation efficiency, which could be detrimental or beneficial depending on the application.

So, the choice of substrate plays a crucial role in determining the antenna’s electrical and mechanical characteristics. When altering the substrate, careful consideration and possible redesign are necessary to maintain or improve the antenna’s performance in the desired application.

In the parts that follow, copper foil has been utilized as a conductive medium for both simulation and fabrication. The optimized dimensions of the antenna are listed in Table 1 (relative permittivity of substrate (paper) $\varepsilon_{r}$ = 3.2, loss tangent = 0.05, and thickness of 0.44 mm). The antenna has an overall dimension of 40 mm × 40 mm × 0.44 mm and has a partial ground plane of 40 mm × 16 mm. There are three radiators i.e., the modified C, a U, and a C shaped. These shapes are made to fit a 50 Ω impedance, and are designed to reach a multiple band. They are fed by a microstrip line. The radiating elements are divided into three sections: the modified C-, U- and C-shaped radiator in Section 1, Section 2, and Section 3, respectively. The U- and C-shaped radiators are connected and disconnected to the main radiator (modified C shaped) using two PIN diodes. Two PIN diode switches are used to connect and separate the radiator’s three portions. Equivalent circuits for the ON and OFF states of the PIN diode ‘D’ are depicted in Figure 2d. A low value resistor (‘R_S_’) and a low value inductor (‘L’), form a simple RL series circuit for the ON state. It is equivalent to an RLC circuit in the OFF state because an inductor (L) is connected in parallel with a high-value resistor (R_S_) and a capacitor (C_R_). A DC bias voltage of +3 V and 0 V, respectively, can be used to turn “ON” and “OFF” the PIN diodes. The diodes utilized are SMP1345-040LF diodes from Skyworks Solutions Inc., which are practical and affordable. The diode has a resistance, inductance, and capacitance of 1 Ω, 045 nH, and 0.2 pF, respectively, in the ON state, and 3.3 kΩ and 0.2 pF, respectively, in the OFF state, according to the data sheet. The parametric study is then performed to optimize the design parameters of the proposed antenna and a simulation is performed using HFSS.

## 3. Fabrication of Paper-Substrate Antenna

Fabricating an antenna using two sheets of photo paper, each with a thickness of 0.22 mm, involves several steps. Below is the fabrication process:

**Step-1** The optimized antenna dimensions as mentioned in Table 1 have been considered for fabrication. Further, a photoresist film is applied to the copper surface. Subsequently, the circuit layout is transferred onto the photoresist using a photomask, a UV light source, and a NaOH solution to remove the unexposed film.

**Step-2** The copper is subjected to wet etching. This process reveals the adhesive layer where the copper has been removed, while the opposite side remains protected by the original adhesive copper tape. In essence, the first two steps closely resemble standard photolithographic techniques. Then, the adhesive-backed copper foil is placed on a clean, flat surface with the adhesive side facing up. Carefully, the backing is peeled off the copper foil to expose the adhesive side.

**Step-3** A sacrificial layer is affixed to the copper side, after which the protective layer is removed. The sacrificial layer serves a crucial role in maintaining the relative distances among layout features, especially when they are not physically connected. This layer can be as simple as a sheet of paper or a plastic adhesive tape. In the former case, the exposed adhesive (where the copper has been removed) provides sufficient adhesion for attaching the sacrificial paper sheet. In the latter case, the adhesion of the sacrificial layer is deliberately chosen to be lower than that of the copper tape itself. The antenna and the ground plane of the antenna are printed on two sheets of photo papers, each with a thickness of 0.22 mm. Two sheets are used to help to reach a reasonable thickness (0.44 mm). Alignment marks have been implemented on the photo papers so that the two photo papers can be glued together with correct alignment to form a single antenna substrate.

**Step-4** The etched metal is transferred onto the hosting paper substrate, and ultimately, the sacrificial layer is detached. This final step also eliminates most of the exposed adhesive material, with the effect being more pronounced when a plastic adhesive tape is employed as the sacrificial layer. The SMA, PIN diodes, resistors, and capacitance are then mounted on the antenna using low-temperature solder pastes and epoxies.

**Step-5** The antenna’s performance is tested using the appropriate testing equipment.

## 4. Parametric Analysis

A parametric study of an antenna can be highly sensitive to physical layout variations due to the intricate relationship between the antenna’s design parameters and its performance characteristics. Here is how this sensitivity arises:

**Electromagnetic interactions:** Antenna design parameters, such as the length, width, and spacing of elements, influence how electromagnetic waves interact with the antenna. Small changes in these dimensions can lead to significant variations in the antenna’s impedance, resonance frequency, and radiation pattern.

**Resonance effects:** Antennas are typically designed to resonate at specific frequencies. Variations in physical dimensions can result in shifts in the resonant frequency, potentially causing the antenna to operate outside of the desired frequency band.

**Radiation pattern:** The physical layout affects the radiation pattern of the antenna. Alterations in element size and spacing can lead to changes in the directionality, gain, and beamwidth of the antenna’s radiation pattern.

**Impedance matching:** Antennas need to be impedance matched to the transmission line or circuit to ensure efficient power transfer. Physical-layout changes can impact the impedance, leading to mismatches and reduced performance.

**Material properties:** The choice of substrate and materials also plays a role in antenna performance. Variations in substrate properties can affect the antenna’s efficiency, bandwidth, and radiation characteristics.

**Manufacturing tolerances:** Real-world fabrication processes have tolerances and variations. Small discrepancies in the manufacturing of antenna elements can accumulate and lead to sensitivity to physical layout changes.

The parametric investigation of S11 has been carried out by altering the ground length (L_g_) and feed width (W_f_).

**Figure 3:**
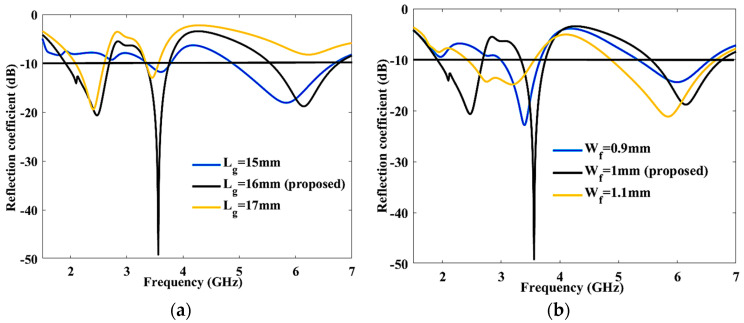
Parametric analysis of proposed antenna (**a**) Ground length (L_g_), (**b**) Feed width (W_f_).

As illustrated in Figure 3a, L_g_ is varied from 15 mm to 17 mm in order to study S11, and 16 mm is found to be the ideal length. Lower-frequency-band response was not reached at L_g_ = 15 mm, while dual-band response was accomplished at L_g_ = 17 mm. When altering the L_g_ within the range of 15–17 mm, it is notable that the antenna’s performance is most affected at L_g_ = 16 mm. This specific dimension results in the optimum return loss, with a peak return loss of 50 dB achieved at 3.6 GHz. However, when L_g_ is adjusted to 15 mm and 17 mm, the return loss becomes significantly lower in certain frequency bands, making it challenging to achieve during the fabrication and testing processes.

Next, W_f_ must be adjusted between 0.9 mm and 1.1 mm, as seen in Figure 3b. When W_f_ is set at 1 mm, the most favorable results are achieved, as this value corresponds to the maximum return loss and encompasses all three frequency bands. However, at W_f_ = 0.9 mm, it is a challenge to achieve a lower band from 1.91 GHz to 2.46 GHz. Conversely, at W_f_ = 1.1 mm, the matching performance is less satisfactory in the lower frequency band.

## 5. Results

**Figure 4:**
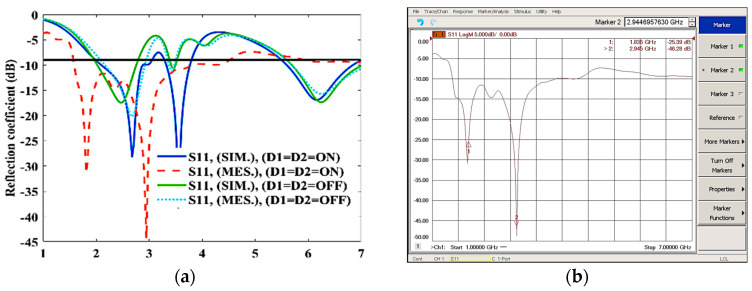

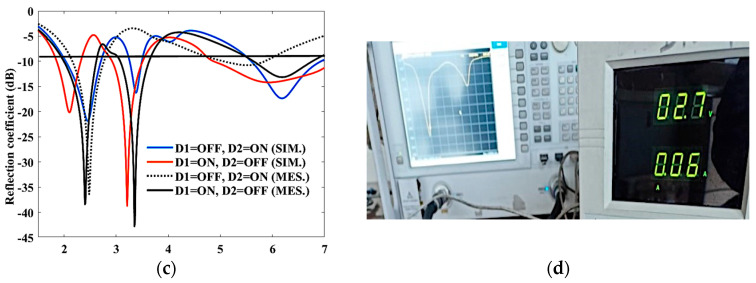
S-parameter characteristics; (**a**) reflection coefficient, when D1 = D2 = ON, OFF; (**b**) measured reflection coefficient, when D1 = D2 = ON; (**c**) reflection coefficient, when D1 = D2 = ON-OFF, OFF-ON; and (**d**) measured reflection coefficient, when D1 = OFF, D2 = ON.

The simulated antenna provides triple-band characteristics ranging 1.91–2.68 GHz, 3.33–3.75 GHz, and 5.55–6.75 GHz. The measured results show wide-band characteristics ranging 1.6–3.82 GHz, when both diodes are in ON states, as shown in Figure 4a,b. When the diodes are in ON-OFF state, as shown in Figure 4c, the antenna provides triple-band characteristics ranging 1.86–2.32 GHz, 2.93–3.5 GHz, and 4.87–6.98 GHz. The measured results show wide-band characteristics ranging 2–2.61 GHz, 3.08–3.63 GHz, and 5.64–6.76 GHz, which is slightly different from the simulated results due to fabrication tolerance.

When the diodes are in the OFF–ON state, as shown in Figure 4c,d, the antenna provides triple-band characteristics ranging 2.04–2.7 GHz, 3.26–3.49 GHz, and 5.6–6.93 GHz, while the measured results show dual-band characteristics ranging 2.16–2.76 GHz and 5.07–5.85 GHz. When both diodes are in OFF states, as shown in Figure 4a, the simulated antenna provides triple-band characteristics ranging 2.03–2.75 GHz, 3.42–3.49 GHz, and 5.7–7.1 GHz, while the measured results show wide-band characteristics ranging 2.17–2.92 GHz, 3.38–3.47 GHz, and 5.7–7.1 GHz.

The simulated results slightly deviate from the measured results due to fabrication tolerance, cable loss, and feeding-probe loss.

**Figure 5:**
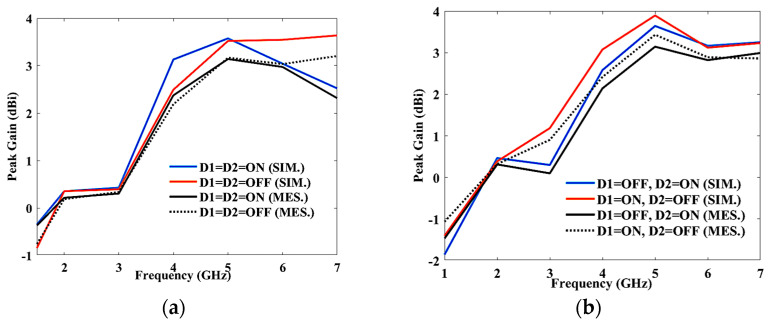

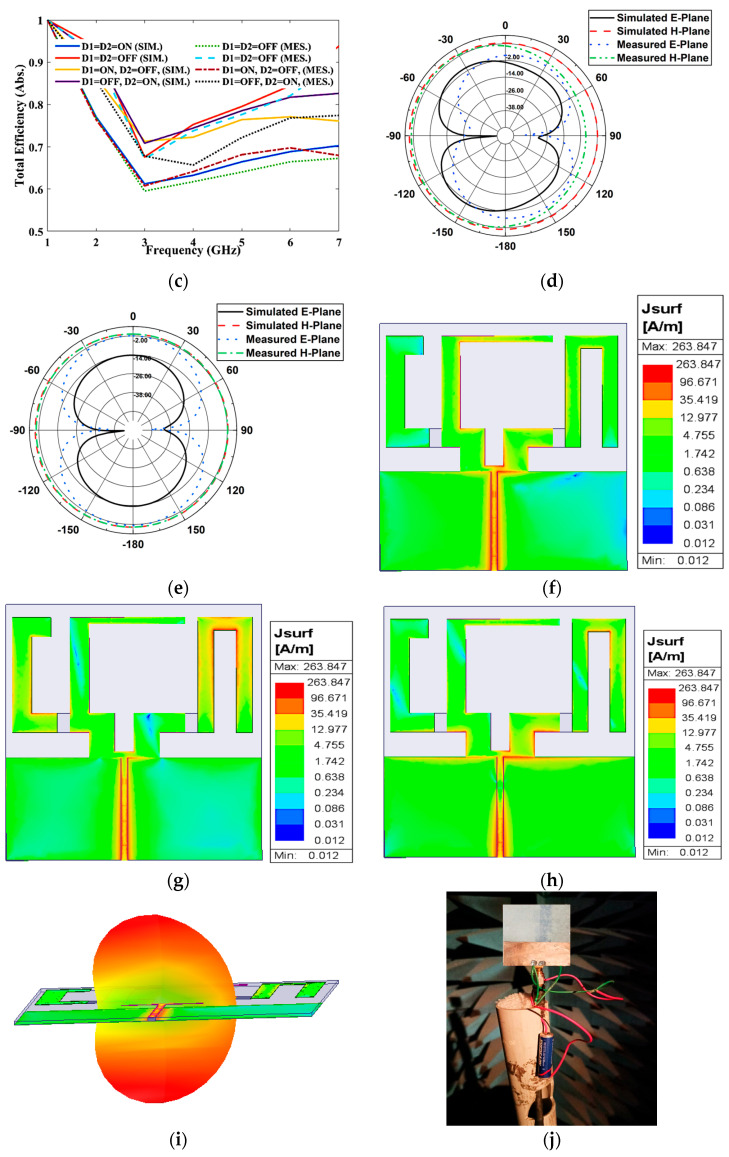
(**a**) Peak gain (D1 = D2 = ON, OFF); (**b**) peak gain (D1 = ON, OFF, D2 = ON, OFF); (**c**) radiation efficiency and E and H plane at (**d**) 3.5 GHz (ON-ON condition) and (**e**) 3.5 GHz (OFF-OFF condition); surface current (**f**) 2.48 GHz, (**g**) 3.5 GHz, and (**h**) 5.6 GHz; (**i**) 3D polar plot at 2.48 GHz; and (**j**) radiation pattern measurement at the ancheoic chamber.

The peak gain for the antenna, with respect to frequency, for different switching characteristics of two diodes is shown in Figure 5a,b. Figure 5a shows the simulated and measured peak gain when the PIN diodes are in “ON-ON” and “OFF-OFF” state. The peak gain lies between 0.35 dBi and 3.57 dBi (simulated), and −0.61 and 3.34 dBi (measured) when both the diodes are ON, and 0.35 and 3.64 dBi (simulated), and 0.68–3.46 dBi (measured) when both the diodes are OFF, respectively. Figure 5b is the simulated and measured peak gain when the PIN diodes are in “ON-OFF” and “OFF-ON” states. The simulated and measured peak gain lies in between 0.38 and 3.89 dBi, and 0.32 and 3.43 dBi when the diodes are ON-OFF, and 0.46 and 3.64 dBi and 0.33 and 3.14 dBi when the diodes are OFF-ON, respectively.

The simulated and measured total efficiency of the antenna is displayed in Figure 5c, in all the different switching condition of the two PIN diodes. Throughout the entire bandwidth, the average efficiency is 60%.

Figure 5d,e shows the 2D pattern for the antenna’s E and H planes for the two different switching states of “ON-ON” and “OFF-OFF”, respectively. The surface current analysis of the proposed antenna at 2.48 GHz, 3.5 GHz, and 5.6 GHz frequencies are displayed in Figure 5f–h. The highest surface current is focused along the feed line and the auxiliary radiator. Figure 5i shows the 3D polar of the proposed antenna at 2.48 GHz, when the diode is in the ON state. The proposed antenna is vertically polarized where theta is varying from −180° to 180° and phi is 0–90°. Figure 5j represents the radiation pattern measurement in the chamber.

### Bending Analysis

**Figure 6:**
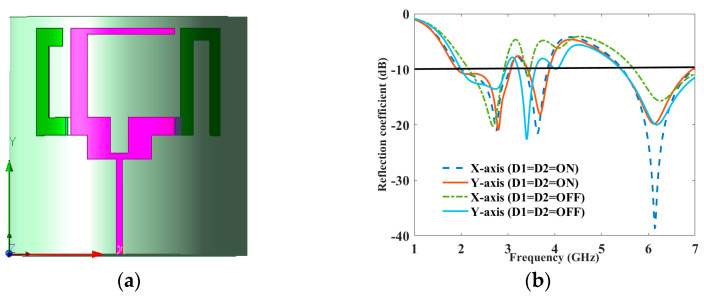
(**a**) Simulated bending and (**b**) reflection coefficient to frequency for bending analysis along the X and Y axis.

In a growing number of wireless applications, versatile antennas are essential components, particularly in contexts like wearable electronics and sensor systems. Nevertheless, the antenna’s performance can be affected when it is bent or flexed from its original configuration, depending on factors such as the antenna design and substrate material. In practical terms, especially in smart-device applications where devices are becoming increasingly compact and thin, it is imperative to assess the antenna’s functionality when positioned on a curved surface. The impedance characteristics of the antenna remain relatively consistent along both X and Y axis when bent. The radiative performance of the antenna, as indicated by S11, remains satisfactory up to a bending angle of 11° in the X axis and 15° in the Y axis for the ON and OFF condition, as depicted in Figure 6.

Table 2 compares the proposed antenna design [P] with the results of earlier research on microstrip antennas on paper substrates. Researchers have already put forth numerous antenna structures with varying sizes and shapes that are beneficial for diverse applications. The proposed antenna achieves a fractional bandwidth of 3.44, which is better than [17,18,19], and slightly less than [20,21], as reported in Table 2. The proposed antenna achieves a gain of 3.4, which is better than the other proposed antenna, but less than [17].

## 6. Conclusions

A low-cost paper substrate is used to design a reconfigurable antenna. Using photo paper as a substrate instead of other substrates decreases the network complexity and makes designing antennas for inkjet printing considerably easier. To increase the bandwidth and add more bands, C- and U-shaped radiators have been used. The antenna was able to function at various frequency bands (1.91–6.75 GHz) by alternating between the primary radiator and the C- and U-shaped radiators using two PIN diodes. The analysis of antenna bending was conducted due to the flexible substrate used in the proposed design. The antenna under consideration demonstrated a peak gain of 3.42 dBi and an efficiency exceeding 60%. As a result, this paper’s substrate antenna is well-suited for effective utilization in mobile bands and multiple wireless applications.

## Figures and Tables

**Table 1:** Optimized antenna dimensions in mm.

L_S_	W_S_	L_f_	W_f_	L_g_	a	b	c	d
40	40	16	1	16	11	4	6	3
e	f	g	h	i	j	k	l	m
8	14	3	1	7	3	3	2.5	2
n	o	p	q					
3.5	2.5	18	1					

**Table 2:** Comparative chart with other antennas.

Ref.	Size (mm^2^)	Frequency Range (GHz)	Efficiency (%)	Substrate Material	Switching Device	Peak Gain (dBi)	Application
[17]	49 × 45	2.3–2.7	43.6	Paper	-	5	RF Electronics
[9]	35 × 40	1.7–4.5	45–58	Paper	-	0.1–1.8	Mobile and wireless
[19]	12 × 37.3	1.53–1.58, 2.75–3.65, 4.25–6.12	55–79	Paper	-	−6, 0–1.8, 2.2	RFID
[18]	30 × 40	1.5–4	50	Paper	PIN diode	2	Wireless
[20]	120 × 60	2–4.27	60–75	Rogers	PIN diode	3–4.2	Wireless
[21]	32 × 36	2.7–5.9	68	FR-4	PIN diode	3.2	Cognitive radio
[22]	38 × 40	2.2, 2.7, 3.8, 5.3	81	Rogers	PIN diode	3.29, 3.5, 2.43, 2.42	5G communication
[23]	58 × 78	3.89–5.9	80	PET	-	3	5G communication
[24]	50 × 40	2.21–5.13	78	Epoxy glass fiber (G-10)	-	2.73–3.74	WLAN, smart wireless
**[Proposed]**	**40 × 40**	**1.91–2.68, 3.3–3.75, 5.55–6.75**	**60–90**	**Paper**	**PIN diode**	**0.12–0.31, 0.28–1.7, 2.82–3.4**	**WLAN, Bluetooth, WiMAX & IoT**

## Data Availability

Data sharing not applicable.
